# Harlequin ichthyosis in a newborn: a rare and severe congenital ichthyosis

**DOI:** 10.11604/pamj.2025.52.174.49375

**Published:** 2025-12-19

**Authors:** Ashish Tamgadge, Kavita Gomase

**Affiliations:** 1 Obstetrical and Gynecological Nursing, Smt Radhikabai Meghe Memorial College of Nursing, Datta Meghe Institute of Medical Science (DU), Wardha, India

**Keywords:** Harlequin ichthyosis, congenital ichthyosis, autosomal recessive disorder

## Image in medicine

A full-term newborn male was delivered via spontaneous vaginal delivery, with a birth weight of 2.9kg. At birth, the neonate presented with multiple dysmorphic features and extensive congenital skin abnormalities. The infant´s entire body showed taut, thickened, parchment-like skin with deep fissures and rigid posturing of the limbs and mouth, consistent with a collodion membrane. The baby had eclabium (eversion of the lips), ectropion (eversion of the eyelids), a flattened nose, and sparse or absent scalp hair. The umbilical cord appeared normal. No other systemic anomalies were noted at the time of initial evaluation. The baby was placed under close monitoring in a neonatal intensive care unit with supportive care, including maintenance of skin hydration, temperature regulation, and prevention of infection. Dermatological evaluation confirmed the clinical diagnosis of harlequin ichthyosis, a rare, severe autosomal recessive disorder of keratinization caused by mutations in the ABCA12 gene. This condition is characterized by defective lipid transport in the epidermis, resulting in abnormal skin barrier formation. Prognosis remains guarded, with neonatal mortality primarily due to sepsis, dehydration, or respiratory failure. Management includes systemic retinoids such as acitretin, which may help accelerate shedding of the collodion membrane and improve long-term outcomes. Genetic counselling was offered to the parents, who had no prior family history of skin disorders or consanguinity.

**Figure 1:**
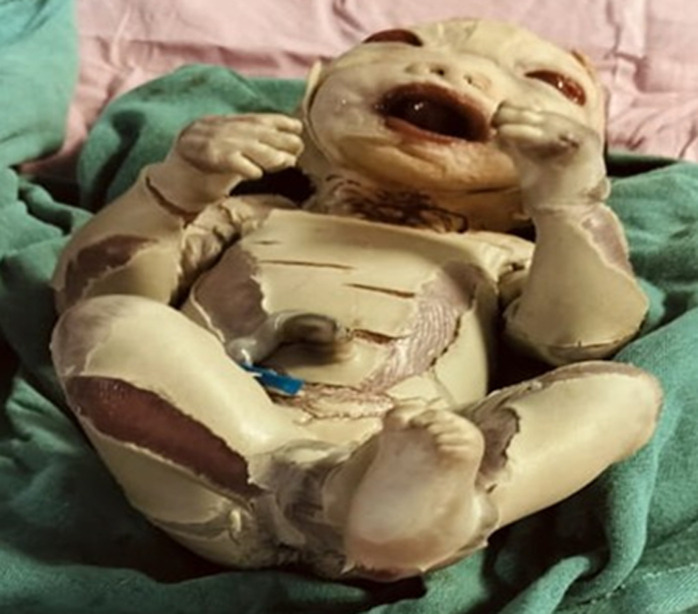
newborn with classic features of harlequin ichthyosis, thickened, fissured skin, eclabium, and ectropion

